# Construction and characterization of two BAC libraries representing a deep-coverage of the genome of chicory (*Cichorium intybus *L., Asteraceae)

**DOI:** 10.1186/1756-0500-3-225

**Published:** 2010-08-11

**Authors:** Lucy Gonthier, Arnaud Bellec, Christelle Blassiau, Elisa Prat, Nicolas Helmstetter, Caroline Rambaud, Brigitte Huss, Theo Hendriks, Hélène Bergès, Marie-Christine Quillet

**Affiliations:** 1 Univ Lille Nord de France, F-59000 Lille, France, Stress Abiotiques et Différenciation des Végétaux Cultivés (SADV), UMR INRA-USTL 1281, Bât. SN2, F-59655 Villeneuve d'Ascq, France; 2 Centre National des Ressources Génomiques Végétales (CNRGV), INRA, Chemin de Borde Rouge, BP 52627, F-31326 Castanet Tolosan, France

## Abstract

**Background:**

The Asteraceae represents an important plant family with respect to the numbers of species present in the wild and used by man. Nonetheless, genomic resources for Asteraceae species are relatively underdeveloped, hampering within species genetic studies as well as comparative genomics studies at the family level. So far, six BAC libraries have been described for the main crops of the family, *i.e*. lettuce and sunflower. Here we present the characterization of BAC libraries of chicory (*Cichorium intybus *L.) constructed from two genotypes differing in traits related to sexual and vegetative reproduction. Resolving the molecular mechanisms underlying traits controlling the reproductive system of chicory is a key determinant for hybrid development, and more generally will provide new insights into these traits, which are poorly investigated so far at the molecular level in Asteraceae.

**Findings:**

Two bacterial artificial chromosome (BAC) libraries, CinS2S2 and CinS1S4, were constructed from *Hin*dIII-digested high molecular weight DNA of the contrasting genotypes C15 and C30.01, respectively. C15 was hermaphrodite, non-embryogenic, and *S*_2_*S*_2 _for the *S*-locus implicated in self-incompatibility, whereas C30.01 was male sterile, embryogenic, and *S*_1_*S*_4_. The CinS2S2 and CinS1S4 libraries contain 89,088 and 81,408 clones. Mean insert sizes of the CinS2S2 and CinS1S4 clones are 90 and 120 kb, respectively, and provide together a coverage of 12.3 haploid genome equivalents. Contamination with mitochondrial and chloroplast DNA sequences was evaluated with four mitochondrial and four chloroplast specific probes, and was estimated to be 0.024% and 1.00% for the CinS2S2 library, and 0.028% and 2.35% for the CinS1S4 library. Using two single copy genes putatively implicated in somatic embryogenesis, screening of both libraries resulted in detection of 12 and 13 positive clones for each gene, in accordance with expected numbers.

**Conclusions:**

This indicated that both BAC libraries are valuable tools for molecular studies in chicory, one goal being the positional cloning of the *S*-locus in this Asteraceae species.

## Introduction

Chicory (*Cichorium intybus *L.) is an Asteraceae (Compositae) species belonging to the Lactuceae tribe of the Lactuoideae subfamily [1,2], as are lettuce and other vegetable crops (*e.g*. endive and salsify), and widespread weeds (*e.g*. dandelion). The genus *Cichorium *includes 6 diploid species (2n = 18) native from the Old World [3]. The closely related species chicory (*C. intybus *L.) and endive (*C. endivia *L.) have been domesticated and are mainly cultivated in Europe. Wild chicory is perennial [3] but the crop has been selected to be cultivated as a biennial species. Four main cultivar groups are distinguished, depending on the use of their roots in transformed products (root or industrial cultivar group) or the consumption of leaves as fresh or cooked vegetables (witloof, pain de sucre, and radicchio cultivar groups) [4]. Though these crops are not highly ranked on the scale of world's agriculture, they are important at the regional level in Belgium, the North of France, and The Netherlands, where the production of root and witloof chicory is concentrated. Similarly, about 85% of the Italian radicchio production is performed in the north eastern part of that country [4].

Wild forms of *C. intybus *have been reported to be strictly allogamous [3], and diallel cross analyses performed in witloof and radicchio cultivar groups showed that selfing is avoided by a strong sporophytic self-incompatibility system controlled by a single multiallelic *S*-locus [5,6]. As for many allogamous species, breeder's work in the early 1960's showed heterosis effects in progenies obtained from crosses between relatively distant selected genotypes, indicating that F_1 _hybrids would be the cultivar type to develop for chicory [7]. Variability for the level of self-fertility was found in different cultivar groups [6,8,9], and in the cultivar group witloof, partly self-compatible genotypes were selected to produce inbred lines used as F_1 _hybrids progenitors [10,11]. Selection pressure for self-fertility was not strongly applied in other cultivar groups.

Resolving the molecular mechanisms underlying traits controlling the reproductive system of chicory is a key determinant for chicory hybrid development, and will more generally provide new insights into these traits, which are poorly understood so far at the molecular level in Asteraceae [12-14]. To achieve these goals, both molecular genetic tools and genomic resources are necessary, and a consensus genetic map based on 472 transferable codominant SSR and SNP markers covering the 9 chromosomes of the *C. intybus *haploid genome was recently established [15]. Furthermore, over 80,000 chicory and endive EST sequences are now publicly available on the NCBI dbEST site [16,17] and the CGPDB site (Compositae Genome Project Database, http://cgpdb.ucdavis.edu/asteraceae_assembly/).

BAC libraries are essential tools for detailed characterisation of genomic regions containing genes of interest by the construction of physical maps and positional cloning. Despite their importance in terms of numbers of species present in the wild, and used by man [2], Asteraceae genomic resources are relatively underdeveloped, hampering the development of within species genetic studies, and comparative genomics studies at the family and clade levels [*e.g*. [18-20]. So far, six BAC libraries have been described for Asteraceae species, and they are restricted to the main crops of the family, *i.e*. lettuce [21] and sunflower [22-25]. Here we present the characterization of BAC libraries of *C. intybus *constructed from two genotypes differing in traits related to sexual reproduction, *i.e*. self-incompatibility and male sterility, and vegetative reproduction, *i.e*. somatic embryogenesis. To demonstrate the utility of these genomic libraries, we used two cDNA probes corresponding to single copy genes putatively involved in somatic embryogenesis to screen the libraries in order to have access to the gene sequences.

## Methods

### Plant materials

Two different *C. intybus *genotypes (C15 and C30.01), selected from a Hungarian landrace population (Koospol), were used for the construction of BAC libraries. C15 was obtained after selfing of the K59 genotype, and is hermaphrodite, non-embryogenic [16], and homozygous *S*_2_*S*_2 _for the *S*-locus. C30.01 was obtained from an open pollination of C30, descendant from K59 upon selfing. C30.01 is male sterile, embryogenic, and heterozygous *S*_1_*S*_4 _at the *S*-locus. *S*-locus genotypes were determined by test crosses; dominance relationships between the *S*-alleles being *S*_1 _= *S*_4_>*S*_2 _for the pistil, and *S*_1_>*S*_4 _>*S*_2 _for the pollen. Both genotypes were *in vitro *propagated, either by organogenesis (C15) [26], or by somatic embryogenesis (C30.01) [27]. Ten clones of each genotype were acclimated in a glasshouse at 20°C, with a 16/8 h light/dark cycle, and grown for about three months. After transferring the plants 5 to 7 days in a dark room, young leaves were collected, frozen in liquid nitrogen and stored at - 80°C before extracting high molecular weight (HMW) genomic DNA.

### BAC libraries construction

HMW DNA of C15 and C30.01 genotypes was prepared as described by Peterson et al. [28] with the following modifications: (1) PVP was lowered to 0.25% w/v in sucrose-based extraction buffer (0.01 M Tris, 0.1 M KCl, 0.01 M EDTA pH 9.4, 500 mM sucrose, 4 mM spermidine, 1 mM spermine tetrahydrochloride, 0.1% w/v ascorbic acid, 0.25% w/v PVP-40, and 0.13% w/v sodium diethyldithiocarbamate) as described in Chalhoub et al. [29], (2) lysis buffer was 1% w/v sodium lauryl sarcosine, 0.3 mg/ml proteinase K and 0.13% w/v sodium diethyldithiocarbamate dissolved in 0.5 M EDTA pH 9.1, (3) after lysis of the nuclei, agarose plugs were pre-washed 1 h in 0.5 M EDTA pH 9.1 at 50°C, 1 h in 0.05 M EDTA pH 8.0 at 4°C, and then stored at 4°C [30]. Embedded HMW DNA was partially digested with *Hin*dIII (New England Biolabs, Ipswich, Massachusetts), subjected to two size selection steps by pulsed-field electrophoresis, using a BioRad CHEF Mapper system (Bio-Rad Laboratories, Hercules, California), and ligated to pIndigoBAC-5 *Hin*dIII-Cloning Ready vector (Epicentre Biotechnologies, Madison, Wisconsin). Pulsed-field migration programs, electrophoresis buffer and ligation desalting conditions were performed for the construction of CinS2S2 library as described in Peterson et al. [28]. These steps of the procedure were modified for the construction of CinS1S4 library, according to Chalhoub et al. [29], in order to increase the insert size (see Results and discussion).

After electroporation of T1 resistant DH10B electrocompetent cells (Invitrogen, Carlsbad, California), transformants were incubated in SOC medium for one hour, and 100 μL of transformants were plated on LB agarose medium containing 12.5 μg/mL chloramphenicol, 80 mg/mL X-gal and 100 mg/mL IPTG for colony counting and insert size estimation. Glycerol stocks (6%, v/v) of the remaining transformants (2.9 mL) were stored at -80°C, for later plating and colony picking. BAC DNA from randomly selected plated colonies of the two libraries was isolated using NucleoSpin fast purification kit (Macherey-Nagel, Düren, Germany). DNA from each BAC was dissolved in 30 μl, and half of this amount was digested with 10 units of *Not*I (New England Biolabs) to excise inserts. The digested BAC DNA was separated in 1% agarose gel by pulsed-field electrophoresis at 12°C in 0.25 × TBE buffer at 6 V/cm with a linear ramp pulse time of 5-15 s for 16 h. Insert sizes were estimated with reference to a Lambda ladder (New England Biolabs).

Colony picking was carried out using a robotic workstation QPix2 XT (Genetix, New Milton, Hampshire, UK) using a white/blue selection. White colonies were arranged in 384-well microtiter plates containing LB medium with chloramphenicol (12.5 μg/mL) supplemented with 6% (v/v) glycerol.

### BAC libraries screening

High-density colony filters were prepared from the complete CinS2S2 and CinS1S4 libraries using a robotic workstation QPix2 XT (Genetix). BAC clones were spotted using a 5 × 5 pattern onto 22 × 22 cm Immobilon-Ny+ filters (Millipore Corporate, Billerica, Massachusetts). On each filter, 27,648 unique clones were spotted in duplicate, and clones were grown at 37°C for 17 h. Filters were then processed as follows: (1) denaturation on Whatman paper soaked with a solution of 0.5 M NaOH and 1.5 M NaCl for 4 min at room temperature, and for 10 min at 100°C, (2) neutralization on Whatman paper soaked with 1 M Tris-HCl pH 7.4, and 1.5 M NaCl for 10 min, incubation in a solution of 0.25 mg/mL proteinase K (Sigma Aldrich, St. Louis, Missouri) for 45 min at 37°C, baking for 45 min at 80°C, and (3) fixation by UV on a Biolink 254 nm crosslinker (Thermo Fischer Scientific, Waltham, Massachusetts) with an energy of 120,000 μJoules. Radiolabelling of probes and hybridization of the filters were performed as described in Sambrook and Russel [31]. Hybridized filters were imaged with a Storm 860 PhosphorImager (GE Healthcare, Little Chalfont, UK), and analyses were performed using the HDFR software (Incogen, Williamsburg, Virginia).

To evaluate the level of contamination by organelle DNA in the libraries, filters were hybridized with four mitochondrial and four chloroplast specific probes generated by PCR using total (*i.e*. nuclear, mitochondrial and chloroplast) DNA from *C. intybus *(genotype K59) as template, and primers described in Table 1. PCR products were purified with the NucleoSpin Extract II kit (Macherey-Nagel). Labelling of the probes with [^32^P]dCTP was performed by random priming using the Ready-To-Go DNA Labelling Beads kit (GE Healthcare), and unincorporated nucleotides were then removed using Illustra ProbeQuant G-50 Micro Columns (GE Healthcare).

**Table 1:** Mitochondrial, chloroplast, and nuclear genes derived probes, and associated primer sequences used for the characterization of the CinS2S2 and CinS1S4 libraries.

**Probe**^***a***^	Description	**Accession numbers**^***b***^	Primer sequences (5' → 3')	Tm (°C)	Size (bp)
*atpA*	F1-F0 ATP synthase, subunit alpha	X80469, X51422, AF034118, X52838	L: gatcttgtcaagcgcactgg R: agtaatgcctgagtcgcagc	66 64	1000
*atp9*	F1-F0 ATP synthase, subunit 9	X51895, DQ539624	L: aataggggccggagctgc R: gaaaggccatcattggggc	69 69	184
*cob*	Apocytochrome b	X98362, EF674047, EF674014, DQ916732	L: gagttatagcagtcctaggg R: ctagtagtaagcaatccgcc	55 58	697
*cox2*	Cytochrome c oxydase, subunit 2	AJ414385, EF547230, DQ004553, EF488904	L: atttcaagacgcagcaacacc R: gtactacctcgtccattgag	65 56	264
*matK*	Maturase K	AJ633132	L: tggttcaggctcttcgctattgg R: cgtcccttttgaagcaagaattg	70 68	398
*ndhF*	NADH dehydrogenase	AY504736	L: tacttgtattgattctatttctttg R: caacaagattaaagattaaaaaag	55 55	581
*rbcL*	Ribulose 1,5-biphosphate carboxylase/oxygenase, large subunit	L13652	L: ttgccgagataatggcctac R: ccaaagatctcggtcagagc	64 64	336
*trnL-trnF*	Intergenic spacer tRNA-Leu (trnL)-tRNA-Phe (trnF) genes	FJ490769	L: ggttcaagtccctctatcccc R: ctaccagctgagctatcccg	65 64	397
*CiAGP *	Arabinogalactan protein	DT212458	L: aaaccaaccaagactttgaccacg R: cccccttaagtttccacaaattac	58 64	418
*CiSTM*	*SHOOT MERSISTEMLESS *transcription factor	GU189066	L: gtaggtacatcatgtttgatggggtttggag R: ccctaccttttggcaattca	73 64	344

^*a*^*atpA*, *atp9*, *cob *and *cox2 *correspond to mitochondrial sequences, *matK*, *ndhF*, *rbcL *and *trnL-trnF *to chloroplast sequences, and *CiAGP *and *CiSTM *to cDNA sequences.^*b *^Multiple accession numbers for a probe indicate that primer pairs were designed from the consensus sequence of these accessions.

Some positive BAC clones detected with mitochondrial specific probes were subjected to a fingerprint analysis. BAC DNA was isolated as described in the previous section, and was digested with 100 units of *Eco*RI (New England Biolabs) in a final volume of 60 μL. Thirty five μL of restriction product were separated in 0.7% agarose gel in 1 × TAE buffer at 80 V for 22 h, and fragments were visualised under UV after ethidium bromide staining. Fragment sizes were estimated with reference to the Raoul™ molecular weight marker (MP Biomedicals, Santa Ana, California).

DNA probes corresponding to chicory genes *CiAGP *(arabinogalactan protein), and Ci*STM *(*SHOOT MERISTEMLESS*, a *KNOTTED1-LIKE *transcription factor) were used to ascertain the utility of the libraries to obtain clones containing single copy genes. The partial cDNA sequence of *CiAGP *was obtained from subtractive EST libraries, constructed from leaf explants of two chicory genotypes differing for their embryogenic capacity when cultivated under somatic embryogenesis-inducing conditions [16]. The full length *CiSTM *cDNA was obtained after amplification with degenerated primers, and RACE PCR of cDNA obtained from leaf explants of the embryogenic interspecific (*C. intybus *× *C. endivia*) genotype 474 [32] cultivated under somatic embryogenesis-inducing conditions (unpublished). *CiAGP *and *CiSTM *probes were generated by PCR from cDNA clones by using specific primers (Table 1). Positive clones detected by hybridization were validated individually by PCR amplification using the primer pairs used for probes synthesis, and visualisation of PCR products after agarose gel electrophoresis.

## Results and discussion

### BAC libraries construction, and general features

**Figure 1:**
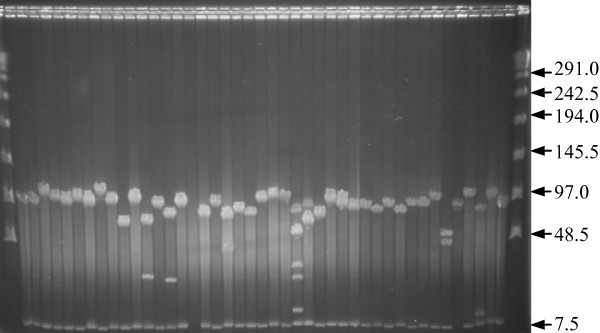
**Insert-size analysis of 43 randomly-picked BAC clones from the CinS2S2 BAC library**. DNA samples were digested with *Not*I and separated on a 1% agarose gel by pulsed-field electrophoresis. Insert sizes were estimated relative to a Lambda DNA size ladder. The 7.5 kb common band is the vector pIndigoBAC-5.

**Figure 2:**
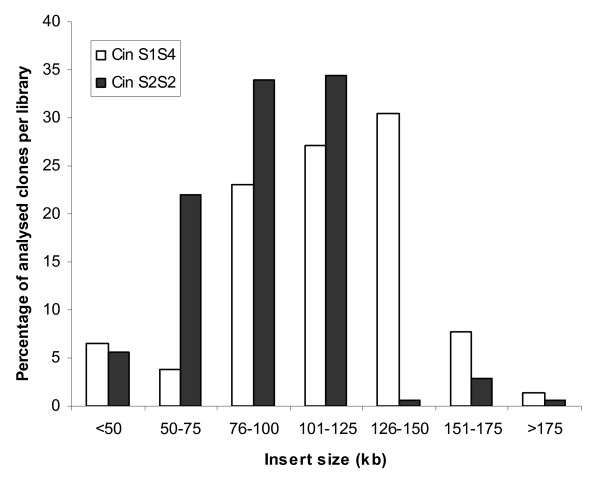
**Insert size distribution of clones randomly selected from the CinS2S2 (178 clones) and the CinS1S4 libraries (303 clones)**.

Two BAC libraries were constructed in pIndigoBAC-5 with *Hin*dIII from DNA of two *C. intybus *genotypes differing in traits related to sexual and vegetative reproduction. Names of the libraries, CinS2S2 and CinS1S4, refer to the *S*-locus genotypes of the plants used to prepare HMW DNA, being S_2_S_2_, for C15, and S_1_S_4 _for C30.01. The main characteristics of the BAC libraries are summarized in Table 2. The CinS2S2 and CinS1S4 libraries consist of 89,088 and 81,408 clones, respectively, arrayed in a total of 444 384-well microtiter plates. A random selection of 178 clones for CinS2S2 and 303 clones for CinS1S4 was analysed after *Not*I digestion, followed by pulsed-field gel electrophoresis (Figure 1). From the clones sampled, the number of clones without insert was 1 (0.56%) and 8 (2.64%) for the CinS2S2 and CinS1S4 libraries, respectively. The average insert sizes of the CinS2S2 and CinS1S4 libraries were calculated to be about 90 kb and 120 kb, respectively (Figure 2). More than 70% of clones from CinS2S2 and 90% for CinS1S4 libraries showed an insert size greater than 75 kb. The higher mean insert size obtained for CinS1S4 library could be explained by modifications of the first sizing step applied for the construction of CinS2S2 library. During the construction of this library, partial digested DNA was separated using the following parameters: 6 V/cm, 18 h, 120°, 1-40 s linear ramping in 0.5 × TBE. For the CinS1S4 library construction performed later on, two programs were aggregated: 6 V/cm, 18 h, 120°, 1-40 s linear ramping in 0.25 × TBE directly followed, in the same gel without removing or handling the DNA, by 5 V/cm, 8 h, 120°, 4-5 s ramping linear in the same buffer as described by Chalhoub et al. [29].

**Table 2:** Characteristics of CinS2S2 and CinS1S4 BAC libraries.

Library	CinS2S2	CinS1S4	Total
No. of clones	89,088	81,408	170,496
No. of 384-well plates	232	212	444
Average insert size (kb)	91.78	118.20	104.40
Empty clones (%)	0.56	2.64	1.55
mtDNA clones (%)	0.02	0.03	0.03
cpDNA clones (%)	1.00	2.35	1.65
Nuclear haploid genome equivalents	5.75	6.53	12.28

### Screening with mitochondrial and chloroplast genome probes

The mitochondrial (mt) genome of chicory has been estimated to be 300-400 kb [33], but genome structure and gene order are unknown. Similarly, in other Asteraceae species, mt genomes are poorly documented, and no complete sequences are publicly available. The probe mixture of four chicory mitochondrial genes (*atpA*, *atp9*, *cob*, and *cox2*) led to the detection of 43 and 29 positive clones in the CinS2S2 and CinS1S4 libraries, respectively. A set of 10 positive clones were randomly selected from each library, and subjected to PCR amplification with primers designed for the mt probes production. Five clones for library CinS2S2 and 8 clones for library CinS1S4 gave PCR products of the expected sizes for at least one of the targeted genes (Table 3). Three clones gave a PCR product only with *cob *primers, and four clones with *atpA*, *atp9 *and *cox2 *primers. The remaining six clones could be amplified with *atpA *primers (1 clone), *atpA *and *cox2 *primers (2 clones), or *atpA *and *atp9 *primers (3 clones). To investigate whether the BAC clones containing the *cob *gene were possibly contiguous with those containing other mt genes, the 13 PCR validated BAC clones were subjected to *Eco*RI digestion for fingerprint analysis. Assuming that bands of the same size estimated after electrophoresis represent identical DNA fragments, pair-wise comparisons of fingerprints revealed that BAC clones of which the inserts contained the *cob *gene shared at least 2 fragments (Table 4). Similarly, at least 3 fragments of the same size were found present in fingerprints of BAC clones with inserts containing two or three other mt genes. The smallest insert (clone 09A06) containing the 3 genes *atpA*, *atp9*, and *cox2*, had an estimated size of 80 kb, delimiting the maximum interval containing these 3 genes (Table 4). However, the fingerprint of this clone did not show any fragment in common with fingerprints of *cob*-containing clones. In contrast, fingerprints of 3 other BAC clones with larger inserts containing the genes *atpA*, *atp9*, and *cox2 *(92M03 and 31K22), or the genes *atpA *and *atp9 *(96A09), shared 2 fragments (9.4 and 11.2 kb, respectively) with fingerprints of *cob*-containing clones (06I01, 26G13, and 25G18) (Table 4), suggesting that the inserts of these BAC clones partly overlapped. Considering all informative fingerprint fragments, the construction of a single contig encompassing the 4 mt genes was only possible when the BAC clone inserts are assumed to originate from a circular mt genome. As only 2 fragments were found in common between the *cob*-containing clones and the other clones, the *cob *gene may be located in a more distant part of the mt genome. Further experiments are necessary to determine the physical distances between these 4 genes. In sunflower, *atpA *and *cob *genes were reported to be only 10 kb apart, in a region associated with cytoplasmic male sterility [34,35]. However, because of frequent genome rearrangements, the organization of mitochondrial genes can vary between related species, and even within a single species [36].

**Table 3:** PCR amplifications of mitochondrial and chloroplast genome specific sequences on ten randomly selected clones of the CinS2S2 and CinS1S4 libraries detected after filter hybridization.

	Mitochondrial genome specific sequences					Chloroplast genome specific sequences				
	**Clone**	***cox2***	***atpA***	***atp9***	***cob***	**Clone**	*matK *2 kb*	*tnrl-trnf *47 kb*	*rbcl *54 kb*	*ndhF *124 kb*

CinS2S2 (90 kb)	06I01	-	-	-	+	02L02	+	-	-	-
	09A06	+	+	+	-	04M20	-	-	-	-
	09E24	-	-	-	-	06I22	+	-	-	+
	09I04	-	-	-	-	12E23	-	+	-	-
	13F07	-	-	-	-	26G24	+	+	+	-
	13M05	-	+	-	-	32H03	-	+	+	+
	26G13	-	-	-	+	37C04	+	+	-	-
	27J01	-	-	-	-	41L23	-	+	+	+
	41H20	+	+	+	-	51H20	-	-	+	-
	54B09	-	-	-	-	61O16	-	+	+	-

CinS1S4 (120 kb)	01H06	+	+	-	-	02N01	-	+	+	+
	16P11	+	+	-	-	21F01	+	-	-	+
	22B14	-	-	-	-	45D02	+	+	+	+
	25G18	-	-	-	+	51D03	-	-	+	+
	27O16	-	+	+	-	55M03	+	+	+	+
	31K22	+	+	+	-	62G03	+	+	+	+
	91G03	-	+	+	-	72G02	-	+	+	+
	92M03	+	+	+	-	84D02	+	-	-	+
	96A09	-	+	+	-	91N03	+	+	+	+
	108A12	-	-	-	-	106P01	+	+	+	+

+ indicates the presence of a PCR product of the expected size after agarose gel electrophoresis;

- indicates no amplification; * Sequence coordinates on *H. annuus *and *L. sativa *chloroplast genome according to [37].

**Table 4:** Fingerprint analysis of BAC clones containing mt genes.

				Informative fingerprint fragment (kb)						
**Library**	**Clone**	**mt gene present**	**Insert size (kb)**	**4.8** **4.9**	**11.2**	**5.6**	**11.7** **9.0** **8.8**	**7.0**	**9.4**	**3.6** **4.4**

CinS2S2	09A06	*cox2, atpA, atp9*	80	-	-	+	+	-	-	-
CinS1S4	96A09	*atpA, atp9*	150	-	-	-	+	+	+	-
CinS1S4	92M03	*cox2, atpA, atp9*	150	-	-	+	+	+	+	-
CinS1S4	31K22	*cox2, atpA, atp9*	95	-	+	+	+	+	-	-

CinS2S2	06I01	*cob*	55	+	+	-	-	-	+	+
CinS2S2	26G13	*cob*	60	+	+	-	-	-	-	+
CinS1S4	25G18	*cob*	25	-	-	-	-	-	+	+

DNA of BAC clones was digested with *Not*I for insert size determination, and with *Eco*RI for fingerprint analysis. Fingerprint fragments ranging from 3 to 12 kb were analysed, and fragments were considered as informative when present in at least 2 clones. Only the results for clones containing at least *atpA *and *atp9*, and sharing a fragment with *cob*-containing clones are shown. Clone 09A06 was included as it contained the smallest insert with the 3 genes *atpA*, *atp9*, and *cox2*. + indicates presence of a fingerprint fragment; - indicates absence of a fingerprint fragment.

Complete sequencing of chloroplast (cp) genomes in *Helianthus annuus *and *Lactuca sativa *indicated that genome size (151.1-152.7 kb), and gene content and order are the same in these two species, representing distantly related subfamilies in Asteraceae [37]. The four sequences (*matK*, *ndhF*, *rbcL *and *trnL-trnF*), chosen to evaluate cp contaminations, are distributed over a region of approximately 120 kb of the consensus cp genome of Asteraceae, the most distant genes (*ndhF *and *rbcL*) being separated by a distance of about 70 kb. The mixture of four probes led to the detection of 994 and 1919 positive clones in the CinS2S2 and CinS1S4 libraries, respectively. PCR validation with specific primers performed on 10 positive clones of each library gave PCR products of expected size for at least one primer pair for 9 clones of the CinS2S2 library, and the 10 clones selected from CinS1S4 library (Table 3). A range of 1-3 (CinS2S2), and 2-4 (CinS1S4) PCR products per clone were detected, and the PCR data confirm the gene order described for the cp genome of *H. annuus *and *L. sativa *[37]. In addition, five of the 10 clones sampled in the CinS1S4 library produced amplification products with the four primer pairs, suggesting that a large continuous fragment of the cp genome is present in these clones.

Based on the proportion of clones validated by PCR, we estimated that 0.024% and 0.028% of the BAC clones would result from mtDNA contamination, and that 1.00% and 2.35% of the clones contain putative cpDNA in CinS2S2 and CinS1S4 libraries, respectively. These results are consistent with: (1) the generally low frequency of mtDNA contamination (0-0.3%) reported in recent plant BAC libraries characterizations [38,39], and (2) the range of commonly observed frequencies (0.3-2.5%) attributed to cpDNA contamination [40,41].

The detected BAC clones containing mtDNA and cpDNA sequences could represent valuable tools as a starting point to investigate the structure of organelle genomes in chicory, particularly the mt genome involved in cytoplasmic male sterility [33,42]. However, the possibility that some of these organelle DNA contaminants actually are the result of horizontal transfer of DNA from mitochondria and/or plastid to the nucleus, as has been observed in most of the plant species studied [43-45], cannot be excluded. Whether large organelle DNA fragments exist in Asteraceae nuclear genomes remains to be determined; chromosome fluorescent *in situ *hybridization experiments [46] using chicory mtDNA- and cpDNA-containing BAC clones would be an approach to investigate this question.

### Theoretical and empirical genome coverage

*C. intybus *has a haploid genome size estimated by flow cytometry to be between 1300 Mb (P. Devaux, personnal communication) and 1400 Mb [47]. After subtraction of non-recombinant and putative organelle DNA contaminant clones, considering a haploid genome size of 1400 Mb, and mean insert sizes of 90 kb for CinS2S2 and 120 kb for CinS1S4, the two libraries represent approximately 5.7 and 6.5 haploid genome equivalents, respectively. Together these two libraries provide a coverage of the *C. intybus *genome of about 12.3-fold (Table 2), resulting in a theoretical probability superior to 0.9999 of recovering any targeted genomic sequence [48]. In comparison, the lettuce BAC library described by Frijters et al. [21] covers about 2 genome equivalents, and the 5 combined sunflower BAC libraries published represent about 20 genome equivalents [22-25]. This indicates that the chicory libraries represent a significant genomic resource to investigate Asteraceae genomes.

To validate empirically the genome coverage calculated for both libraries, BAC clones were screened with two cDNA probes (*CiAGP *and *CiSTM*). Mapping data for each of the corresponding genes [49] revealed a unique locus position in the chicory map of Cadalen et al. [15], indicating that they represent single copy sequences. Hybridization allowed the identification of 13 positive clones for *CiAGP*: 2 clones in the CinS2S2 library and 11 clones in the CinS1S4 library. The *CiSTM *probe revealed 5 positive clones in the CinS2S2 library and 7 positive clones in the CinS1S4 library, *i.e*. a total of 12 clones in the combined libraries. The clones detected by hybridization were subjected to PCR amplification with the primer pairs used for the synthesis of the hybridization probes, and agarose gel electrophoresis of PCR products showed amplicons of the expected size for all of them. As for the *CiAGP *gene, an under- and over-representation was found in the CinS2S2 and CinS1S4 libraries, respectively. Further screening with a larger set of single copy sequence probes are necessary to determine if significant differences between libraries exist as for their content in single copy sequences. Nonetheless, experimental results are in accordance with the theoretical genome coverage estimated for the combined libraries, indicating that they constitute an efficient tool to recover single copy genes or genomic sequences for chicory.

### Perspective

One future use of the *C. intybus *BAC libraries will be the positional cloning of the *S*-locus in this species. As in some other plant families, Asteraceae species exhibit a sporophytic self-incompatibility (SSI) system where the pollen phenotype depends on the genotype of the plant that produced the pollen [50]. The pollen and stigma determinants of SSI have been characterised in *Brassica *[51,52], and molecular and genetic studies showed that these genes are different from those involved in the SSI response in Convolvulaceae and Asteraceae, suggesting that different molecular mechanisms of SSI have evolved independently [12,53]. More recently, candidate genes with unknown functions have been identified in *Ipomoeae trifida *(Convolvulaceae) through a positional cloning approach [54]. Similarly, we have chosen for genetic and subsequent physical mapping of the *S*-locus in chicory to identify pollen and stigma determinants of SSI in this Asteraceae species.

### Library avaibility

The libraries described are accessible for scientific research based on collaboration with the laboratories represented by the corresponding authors.

## Competing interests

The authors declare that they have no competing interests.

## Authors' contributions

The project was conceived and supervised by MCQ and TH. LG, AB, CB, and NH realized the construction of the two BAC libraries under the supervision of HB in the CNRGV - INRA, Toulouse. EP performed the hybridizations using probes provided by CR and BH, and NH realized the fingerprint experiment. All authors contributed to the analyses and interpretations of the results, took part in the redaction of the manuscript, and approve its final version.
